# Occurrence and Sources of Triterpenoid Methyl Ethers and Acetates in Sediments of the Cross-River System, Southeast Nigeria

**DOI:** 10.1155/2010/502076

**Published:** 2010-04-18

**Authors:** Orok E. Oyo-Ita, Bassey O. Ekpo, Daniel R. Oros, Bernd R. T. Simoneit

**Affiliations:** ^1^Department of Pure and Applied Chemistry, University of Calabar, P.M.B. 1115 Calabar, Nigeria; ^2^Consultant 72 Marina Lakes Drive, Richmond, CA 94804, USA; ^3^Department of Chemistry, Oregon State University, Corvallis, OR 97331, USA; ^4^COGER, King Saud University, Riyadh 11451, Saudi Arabia

## Abstract

Pentacyclic triterpenol methyl ethers (PTMEs), germanicol methyl ether (miliacin), 3-methoxyfern-9(11)-ene (arundoin), *β*-amyrin methyl ether (*iso*-sawamilletin), and 3-methoxytaraxer-14-ene (sawamilletin or crusgallin) were characterized in surface sediments of the Cross-River system using gas chromatography-mass spectrometry (GC-MS). Triterpenol esters (mainly *α*- and *β*-amyrinyl acetates and hexanoates, and lupeyl acetate and hexanoate) were also found. These distinct compounds are useful for assessing diagenesis that can occur during river transport of organic detritus. Poaceae, mainly Gramineae and *Elaeis guineensis* higher plant species, are proposed as primary sources for the PTMEs and esters in the sediments. PTMEs are biomarkers of specific higher plant subspecies, while the triterpenol esters are indicators of early diagenetic alteration of higher plant detritus.

## 1. Introduction

Pentacyclic triterpenoids have generally been utilized as biomarkers to trace genetic sources of organic matter in sedimentary environments, petroleum exploration, or paleoenvironmental reconstructions of biome changes that document climate change [1–4]. The oleananes, ursanes, fernanes, lupanes, and their derivatives, widely distributed mainly as the oxygenated forms in many varieties of higher plant species, belong to this class of compounds [4–10]. Their characterization in chemotaxonomic studies can provide key information of flora changes [11]. Their tendencies to also resist biodegradation and occurrence in sediments suggest the potential application as specific higher plant derived biomarkers [9, 10]. However, reports of pentacyclic triterpenol methyl ethers (PTMEs) in sedimentary environments are limited to lakes [2, 4, 12]. Other reports have assessed sedimentary input of terrestrial and/or planktonic organic matter with triterpenoid natural products (e.g., [13–15]) and biomass source tracers to smoke aerosols (e.g., burning of sugar cane [16]). Reports of triterpenoid esters in sediments are also limited, because typical extract analyses generally involved saponification as a preparative step. Plant wax analyses without hydrolysis do reveal triterpenol esters as part of the wax esters in epicuticular waxes (e.g., [17, 18]).

Under aerobic conditions, the transformation of plant-derived triterpenoids often involves oxidation, dehydration, hydrolysis, decarboxylation, ring opening, and aromatization reactions [19]. For instance, in coal forming environments, higher plant triterpenoids generally undergo aromatization starting from ring A, triggered by the elimination of the oxygenated functionality at C-3 and proceeding to rings D/E [20–22]. The PTMEs, which are natural products, appear to be more resistant to environmental alteration than the triterpenol esters and thus may be good biomarkers. Triterpenol esters, on the other hand, may be useful for assessing early diagenesis (i.e., hydrolysis) of terrestrial higher plant detritus during river transport. It is the aim of this paper to report on the characterization, occurrence, and sources of PTMEs and triterpenol esters in surface sediments of the Cross-River system, Nigeria. These are minor compounds occurring with the dominant triterpenoids such as taraxerol, amyrin, and lupeol.

## 2. Locale Description

**Figure 1:**
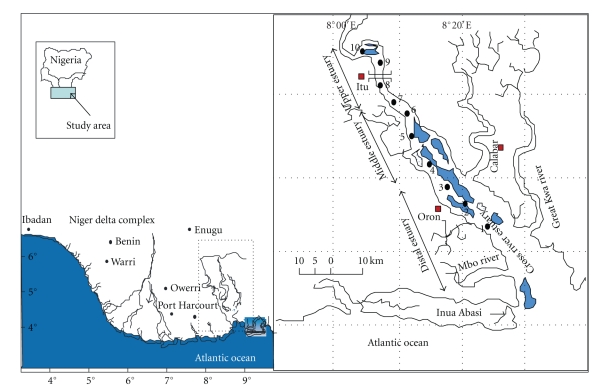
Map of the southeastern part of the Niger Delta of Nigeria showing the sampling locations in the Cross-River estuary.

 The characteristic features of the study area are summarized in Table 1 and the sampling locations of surface sediments are shown in Figure 1. The Cross-River system is one of the largest estuaries located in the eastern edge of the Niger Delta. The whole Cross-River system lies approximately between longitudes 2° 03′ E and 10° 00′ E and latitudes 4° 00′ N and 8° 00′ N and covers an area of 54,000 km^2^, of which 14,000 km^2^ lies in Cameroon and 39,000 km^2^ lies in Nigeria. The river is formed from numerous tributaries arising from the western slopes of the Cameroon Mountains. It flows southwest into the Atlantic Ocean with a discharge rate between 879 and 2533 m^3^/sec [23]. The system is exposed to temporal flooding depending on the tides and the season (wet versus dry) and has large fluctuations in hydrographic conditions [23]. The river system is characterized by the interaction of an estuarine and freshwater-seawater frontal system seaward of the river mouth (typical of a deltaic coastal region) with tidal and wind-driven surface currents. Previous studies focused mainly on fisheries [24], ecology [25], water quality [26], hydrology of the lower Cross-River [23], and hydrocarbons in sediments [27, 28].

## 3. Experimental Methods

Sampling stations were chosen to cover the characteristic features of the river environment as summarized in Table 1. Sediments were collected in January 1999 (dry season) with a Van Veen grab sampler (0.1 m^2^), wrapped with aluminum foil and stored frozen at −4°C until analysis. Freeze-dried sediments were grounded in a disc mill and subsequently sieved to pass 230 mesh to obtain the <63 *μ*m fraction. Extraction and fractionation of the <63 *μ*m fraction were as previously reported by Ekpo et al. [29]. Briefly, 50 g dry samples were extracted in a Soxhlet apparatus with dichloromethane and methanol (2 : 1). Extracts were concentrated, desulfurized (activated Cu), and fractionated by column chromatography on activated silica and alumina. The saturated fraction (F1) was eluted with hexane, the aromatic fraction (F2) with dichloromethane, and the nitrogen-sulfur-oxygen (NSO) containing polar fraction (F3) with dichloromethane-methanol. All fractions were reduced in volume and dried aliquots were weighed for quantitation. Total organic carbon (TOC) analyses for all sediment samples were obtained using an LECO C-S-444 analyser. 

Gas chromatography-mass spectrometry (GC-MS) analyses of the isolated fractions were performed on a Hewlett-Packard Model 6890 GC coupled to a Hewlett-Packard Model 5973 quadrupole MSD. Separation was achieved on a DB5-MS column (30 m × 0.25 mm i.d., 0.25 *μ*m film thickness). The GC operating conditions were as follows. Temperature holds at 65°C for 2 minutes, increases from 65 to 300°C at a rate of 6°C min^−1^, and with final isothermal holds at 300°C for 20 minutes. Helium was used as carrier gas. The sample was injected in the splitless mode with the injector temperature at 300°C. The mass spectrometer was operated in the electron impact mode at 70 eV ionization energy and scanned from 50 to 650 Dalton. Data were acquired and processed using ChemStation software. Compounds were identified by comparison with literature data and interpretation of mass spectrometric fragmentation patterns.

## 4. Results and Discussion

 The analytical results are presented in Table 1. The percentage of total organic carbon (TOC) contents in the sediments ranged between 1.3% and 4.6%, while the extractable organic matter (EOM) ranged between 1.1 and 4.1 g/kg dry weight (dw). The total hydrocarbons determined from F1 and F2 fractions showed the lowest concentration range in sediments from the upper river region (range 2–44 mg/kg dw), a moderate concentration range in sediments from the middle region (range 60–148 mg/kg dw), and the highest concentration range in sediments from the lower deltaic region of the estuary (185–511 mg/kg dw) (Table 1). The data for the Upper Calabar River and Great Kwa River are given as ranges and averages (Table 1). 

### 4.1. Mass Spectrometry

**Figure 2:**
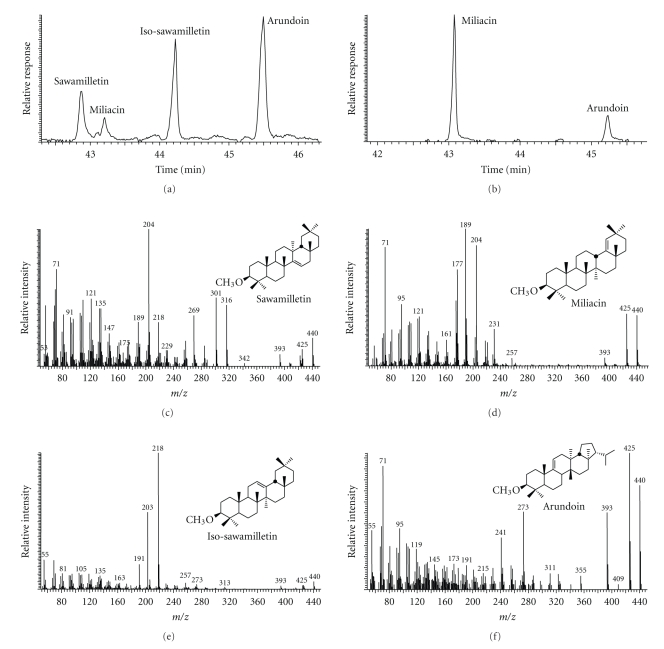
Examples of GC-MS data for triterpenoid methyl ethers in sediment extracts: (a) *m/z* 440 mass fragmentogram for sample GKR-08, (b) *m/z* 440 mass fragmentogram for sample GKR-02, (c) mass spectrum of sawamilletin, (d) mass spectrum of miliacin, (e) mass spectrum of *iso*-sawamilletin, and (f) mass spectrum of arundoin.

Pentacyclic triterpenol methyl ethers (PTMEs) in the bottom sediments were monitored with the *m/z* 440 key ion (M^.+^) in the MS data. Examples are shown in Figure 2 and the mass spectra of the major PTMEs are also given. Their concentrations range from 0.02 to 2.4 mg/kg dw (Table 1). The fragment ion at *m/z* 408 (M-32, minor) and *m/z* 393 (M-15-32) indicates loss of the methoxy group as methanol during fragmentation [30]. Compound 1 has a base peak at *m/z* 189 and accompanying ions at *m/z* 177 and 204, which are characteristic for germanicol (olean-18-en-3*β*-ol). Thus the mass spectrum fits with the methyl ether of germanicol (miliacin). Compound 2 has a base peak at *m/z* 204 which is from the D/E ring of taraxerene after retro-Diels-Alder rearrangement [31]. The additional ions at *m/z* 316, 301, 284, 269, 257, 218, and 189 are characteristic for taraxerol methyl ether. Compound 3 exhibits a different fragmentation pattern, with significant fragments at *m/z* 425 (base peak), 393, 273, 241, and 71. The prominent fragment ion at *m/z* 393 (M-15-32) indicates loss of a methyl group followed by methanol, and *m/z* 273 (M-167) fission of ring C/D and loss of the ring E moiety with the isopropyl group, typical of the fernene type and is assigned as fern-9(11)-en-3*β*-ol methyl ether [32]. The mass spectrum of compound 4 has a base peak at *m/z* 218 and intense ions at *m/z* 191 and 203, which, with the M^.+^ at *m/z* 440 and the typical fragments at *m/z* 425, 408, and 393 indicate 3*β*-methoxyolean-12-ene (*β*-amyrin methyl ether or *iso*-sawamilletin). These mass spectra are also good fits with those reported by Jacob et al. [4]. 

**Figure 3:**
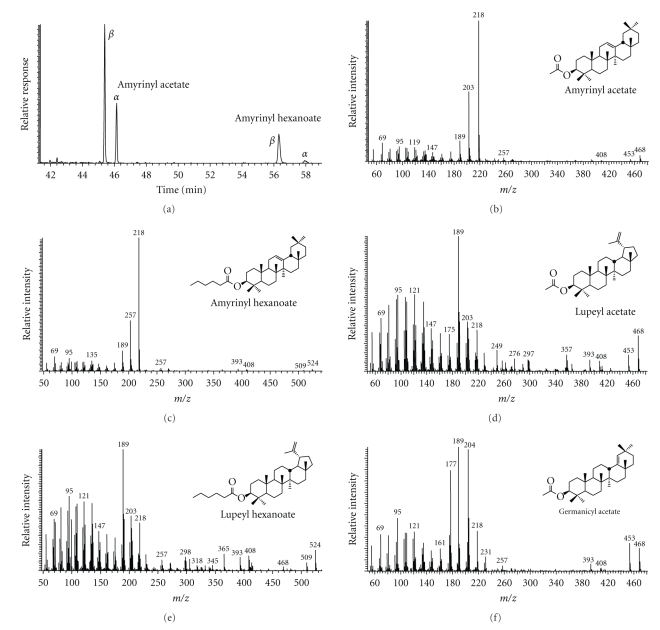
Examples of GC-MS data for triterpenoid esters in sediment extracts: (a) *m/z* 218 mass fragmentogram for amyrinyl esters, (b) mass spectrum of *β*-amyrinyl acetate, (c) mass spectrum of *β*-amyrinyl hexanoate, (d) mass spectrum of lupeyl acetate, (e) mass spectrum of lupeyl hexanoate, and (f) mass spectrum of germanicyl acetate.

 Examples of MS data for the triterpenol esters are shown in Figure 3. The mass spectra of the esters are quite simple, reflecting the fragmentation pattern of the triterpane skeleton with minor ions from the additional acid moiety [33]. Thus, the key ion for the amyrin esters is *m/z* 218 and the mass spectrum of *β*-amyrinyl acetate consists of the M^.+^ at *m/z* 468, loss of CH_3_ to *m/z* 453, and loss of the acetic acid after H transfer to *m/z* 408 (Figures 3(a) and 3(b), resp.). The amyrinyl hexanoates (e.g., Figure 3(c) for 3*β*-isomer) have M^.+^ at *m/z* 524, followed by loss of CH_3_ or the acid moiety to *m/z* 509 and 408, respectively. The mass spectra of lupeyl acetate (Figure 3(d)) and lupeyl hexanoate (Figure 3(e)) also exhibit the dominant fragmentation pattern of the lupene skeleton and the acid moiety is reflected in the M^.+^, M-CH_3_, and M-acid ions. The mass spectrum of germanicyl acetate (Figure 3(f)) has the characteristic fragments for oleana-2,18-diene with significant M^.+^ at *m/z* 468, and M-CH_3_ to *m*/*z* 453 and a minor loss of acetic acid to *m/z* 408. The concentrations of the triterpenol esters range from not detectable to a total of 7.2 mg/kg (Table 1).

### 4.2. Sources and Fate

The detection of these PTMEs in relatively few sedimentary environments may be linked to variation in environmental conditions such as seasonal and environmental differences that determine the biosynthesis of these compounds in specialized tissues of certain species of higher plants [4]. Nevertheless, the PTMEs are natural products introduced directly to the river in organic detritus, probably in leaf litter. The primary sources of PTMEs in this estuary may be from monocotyledonous plants belonging to the Gramineae, on the basis of taxonomic identifications in the vicinity of the study area. According to Jacob et al. [4] plants belonging to the Poaceae produce *iso*-sawamilletin, miliacin, arundoin, and sawamilletin and thus could also contribute to the sources of PTMEs in these sediments. For instance, the occurrence of nine PTMEs from numerous species of Gramineae has been reported (e.g., [30, 34]) and arundoin was found in palm trees, *Elaeis guineensis* [35], and most Poaceae reviewed by Jacob et al. [4].

The persistence of these PTMEs in this estuary may reflect their relative stability to aerobic degradation. We see no evidence that these PTMEs have undergone diagenetic transformations to the 3*α*-PTMEs [4] in the sediments. Taraxerone also reported for reference (Table 1) was detected at almost all the sampling stations. It is a product from the aerobic oxidation of taraxerol from mainly a mangrove origin and a major natural product in most of these samples (Table 1) (e.g., [6, 36–38]). Thus, the relative capacity of these PTMEs to resist biodegradation may enhance their utility as biomarkers for source correlations of specific higher plant subspecies in environmental samples.

 The triterpenol esters in these sediments are mainly acetates and lesser amounts of hexanoates (Table 1). However, based on previous studies of triterpenoid esters in vegetation and sediments (palmitates, stearates, etc., [17, 18]), it is possible that there are even higher molecular weight esters present in these sediments. They are known to elute at much higher GC temperatures and are not detectable by this analytical protocol. The concentrations of the esters are low compared to their parent triterpenols, so a mass balance is not feasible. Nevertheless, their presence in certain sediments may indicate a close input source to that locale, because acetates, like the wax esters, are susceptible to hydrolysis during river transport. The concentrations of the acetates are always 2 to 10 times greater than the hexanoates (e.g., Figure 3(a), Table 1). The highest amounts are observed in the upriver locales and the lowest amounts downriver and in the mangrove bounded estuary. Thus, their source is likely in litter and terrestrial detritus from the grasslands and the deciduous forests and not the mangrove stands.

## 5. Conclusion

Pentacyclic triterpenol derivatives, as the methyl ethers (PTMEs) and alkanoates, were characterized in the sediments of the Cross-River system. The PTMEs that were characterized included germanicol methyl ether (miliacin), 3*β*-methoxyfern-9(11)-ene (arundoin), *β*-amyrin methyl ether (*iso*-sawamilletin), and 3*β*-methoxytaraxer-14-ene (sawamilletin), while the alkanoates consisted mainly of *α*- and *β*-amyrinyl and lupeyl acetates and hexanoates. These distinct biomarkers are readily extractable from river sediments using polar solvent extraction techniques and are identifiable with routine GC-MS analysis by their characteristic mass spectrometric fragmentation pattern. Pentacyclic triperpenol natural products, their derivatives, and degradation products are excellent chemical metrics for extrapolating the impacts that the river/estuary environment imparts on higher plant organic matter.

## Figures and Tables

**Table 1:** Sediment samples, environmental characteristics, and triterpenoid markers in the extracts from the Cross-River estuary, Nigeria.

Zones			I				II			III			IV	V
Sample code			CR-1	CR-2	CR-3	CR-4	CR-5	CR-6	CR-7	CR-8	CR-9	CR-10	UCR 1–10, range (ave.)	GKR 1–11, range (ave.)

Location name			Oron beach				Oku Iboku beach			Itu beach			Upper Calabar River	Great Kwa River

Sample coordinates			4°43.961N 8°21.327E	4°46.531N 8°18.908E	4°49.927N 8°15.501E	4°52.675N 8°12.742E	4°56.879N 8°09.334E	5°00.437N 8°07.062E	5°04.318N 8°06.250E	5°08.198N 8°04.303E	5°12.726N 8°03.491E	5°12.258N 8°00.222E		
Environmental characteristics			*Nypa frutican* with sparsely distributed *Elaeis guineensis* and *Andropogoneae *				*Rizophoria* with sparsely distributed *Elaeis guineensis* and *Andropogoneae *			*Avicennia* with sparsely distributed *Elaeis guineensis* and *Andropogoneae *				

TOC (%)			4.03	3.64	4.35	4.56	4.20	4.38	2.77	1.27	2.66	4.26	na	na
EOM (mg/kg)			3000	3680	1920	2950	3710	4140	2650	1510	1140	1850	na	na
THC (mg/kg)			329	511	227	185	148	60	99	44	16	2	na	na

Compounds (mg/kg dw)	MW	Formula												

Taraxer-14-en-3-one	424	C_30_H_48_O	1.9	2.63	9.6	1.34	3.8	1.1	1.05	0.3	0.39	1.36	0.3–12.8 (4.9)	0.15–21.4 (10.7)

Taraxer-14-en-3*β*-ol methyl ether	440	C_31_H_52_O	0.16	0.21	0.14	0.18	0.51	0.18	0.28	0.26	0.88	0.97	0.2–1.2 (0.4)	0.1–0.6 (0.25)

Germanicol methyl ether	440	C_31_H_52_O	0.05	0.11	0.07	0.06	0.17	nd	0.42	0.11	0.40	0.52	0.3–2.1 (0.7)	0.04–1.07 (0.29)

*β*-Amyrin methyl ether	440	C_31_H_52_O	0.12	0.17	0.04	0.06	0.04	0.08	0.32	0.18	0.22	0.36	nd	0.26–1.80 (0.44)

Fern-9(11)-en-3*β*-ol methyl ether	440	C_31_H_52_O	0.30	0.32	0.31	0.38	0.21	0.22	nd	0.12	0.38	0.94	0.4–2.2 (1.1)	0.01–2.35 (0.68)

*α* *β*-Amyrinyl acetates	468	C_32_H_52_O_2_	0.57	4.73	1.60	0.06	0.15	0.02	0.59	0.31	3.12	0.97	0.8–2.1 (0.9)	0.13–1.05 (0.23)

Lupeyl acetate	468	C_32_H_52_O_2_	0.19	1.31	nd	nd	nd	nd	nd	nd	3.05	nd	nd	nd

Germanicyl acetate	468	C_32_H_52_O_2_	0.11	0.86	0.05	nd	nd	nd	nd	nd	0.18	0.02	nd	nd

*α* *β*-Amyrinyl hexanoates	524	C_36_H_60_O_2_	nd	0.32	0.48	0.03	nd	nd	nd	nd	0.21	0.03	nd	nd

Lupeyl hexanoate	524	C_36_H_60_O_2_	nd	0.02	0.07	0.01	nd	nd	nd	nd	0.02	nd	nd	nd

Abbreviations: EOM: extractable organic matter; THC: total hydrocarbons; nd: not detected (minimum detection limit = 0.002 mg/kg); na: not analyzed.
